# Personality functioning and the pathogenic effect of childhood maltreatment in a high-risk sample

**DOI:** 10.1186/s13034-022-00527-1

**Published:** 2022-11-30

**Authors:** Delfine d’Huart, Joost Hutsebaut, Süheyla Seker, Marc Schmid, Klaus Schmeck, David Bürgin, Cyril Boonmann

**Affiliations:** 1 Department of Child and Adolescent Psychiatric Research, Psychiatric University Hospitals Basel, Basel, Switzerland; 2 Department of Medical and Clinical Psychology, Tilburg University, Tilburg, The Netherlands; 3 Department for Child and Adolescent Psychiatry/Psychotherapy, University of Ulm, Ulm, Germany; 4 LUMC Curium – Department of Child and Adolescent Psychiatry, Leiden University Medical Center, Leiden, The Netherlands; 5 Viersprong Institute for Studies on Personality Disorders, De Viersprong, Halsteren, The Netherlands

**Keywords:** Personality functioning, Self-functioning, Childhood maltreatment, Emotional neglect, Mental health problems, Mediation analysis

## Abstract

**Background:**

While the psychopathological sequalae of childhood maltreatment are widely acknowledged, less is known about the underlying pathways by which childhood maltreatment might lead to an increased risk for mental health problems. Recent studies indicated that impaired personality functioning might mediate this relationship. The aim of the present paper was to extend the current literature by investigating the mediating effect of impaired personality functioning between different types of childhood maltreatment and self-reported mental health problems in a high-risk sample.

**Methods:**

Overall, 173 young adults (mean age = of 26.61 years; *SD* = 3.27) with a history of residential child welfare and juvenile justice placements in Switzerland were included in the current study. The Childhood Trauma Questionnaire (CTQ-SF), Semi-structured Interview for Personality Functioning DSM-5 (STiP-5.1) and the self-report questionnaires of the Achenbach System of Empirically Based Assessment scales (ASEBA) were used. Mediation analyses were conducted through structural equation modeling.

**Results:**

Overall, 76.3% (N = 132) participants indicated at least one type of childhood maltreatment, with emotional neglect being most commonly reported (60.7%). A total of 30.6% (N = 53) participants self-reported mental health problems. Emotional abuse (r = 0.34; *p* < .001) and neglect (r = 0.28; *p* < .001) were found to be most strongly associated with mental health problems. In addition, impaired personality functioning was fond to be a significant mediator for overall childhood maltreatment (*β* = 0.089; *p* = 0.008) and emotional neglect (*β* = 0.077; *p* = 0.016). Finally, impaired self-functioning was found to be a significant mediator when both self-functioning and interpersonal functioning were included as potential mediators in the relationship between overall childhood maltreatment (*β*_*1*_ = 0.177, *p*_*1*_ = 0.007) and emotional neglect (*β*_*1*_ = 0.173, *p*_*1*_ = 0.003).

**Conclusion:**

Emotional neglect may be particularly important in the context of childhood maltreatment, personality functioning, and mental health problems and, therefore, should not be overlooked next to the more “obvious” forms of childhood maltreatment. Combining interventions designed for personality functioning with trauma-informed practices in standard mental health services might counteract the psychopathological outcomes of maltreated children and adolescents.

**Supplementary Information:**

The online version contains supplementary material available at 10.1186/s13034-022-00527-1.

## Introduction

Childhood maltreatment (i.e., emotional neglect, physical neglect, emotional abuse, physical abuse, and sexual abuse) is a major concern that substantially affects millions of people and has been shown to be significantly associated with poor mental health [1, 2]. In a recent review of several meta-analyses of the sequelae of childhood maltreatment, an increased risk for psychopathology was identified as one of the five hallmarks (i.e., increased risk of obesity; increased risk of high-risk sexual behaviors, increased risk of smoking, and increased risk of child maltreatment in children with disabilities) of childhood maltreatment [3]. Although the association between childhood maltreatment and psychopathology is well researched, less is known about the causal relationships and the underlying pathways by which childhood maltreatment might lead to an increased risk for mental health problems. Interestingly, Lang et al. [3] identified resilience as a potential sixth hallmark, given the frequent observation that in all studies some affected individuals seemed to survive without notable consequences. The issue of resilience may point to the potential role of personality and personality functioning as mediators that may partially explain why some individuals experience a much higher burden following childhood maltreatment compared to others.

Childhood maltreatment has often been observed as a precursor related to the onset of personality disorders (PDs), most specifically borderline personality disorder (BPD). Patients with BPD have been found to be almost 14 times more likely to report a history of childhood maltreatment than non-clinical controls, with emotional abuse and neglect being the most prevalent types of childhood maltreatment [4]. Indeed, numerous studies have indicated that exposure to childhood maltreatment is related to various BPD symptoms, such as affective instability, interpersonal problems, identity problems, impulsivity, and suicidal behavior [5–8]. In a community-based study, Brown et al. [9] for instance, reported significantly more childhood maltreatment in participants engaging in Non-Suicidal Self-Injury (NSSI) compared to healthy controls, with emotional abuse and neglect being etiologically more directly associated with self-harm than physical and sexual abuse. The meta-analysis from Liu et al. [10], in addition, found general support for a positive association between childhood maltreatment and impulsivity, with pooled effect sizes ranging from small (i.e., sexual abuse) to large (i.e., emotional abuse). Taken together, studies show an increased risk for PDs and associated symptomatology.

Recently, new perspectives on PDs have been formulated in the fifth edition of the Diagnostic and Statistical Manual of Mental Disorders (DSM-5; [11]) as well as in the 11th edition of the International Classification of Diseases (ICD-11; [12]), which may enable us to approach the role of PDs somewhat differently. Whereas in classic studies, features of PDs are typically seen as a potential symptomatic outcome of childhood maltreatment, newer models rather approach PDs in terms of structural impairments in personality functioning [13]. This reflects an important paradigm shift in which PDs are being seen as dispositions of vulnerability, while traditional PD symptoms, like self-harm or frantic efforts to avoid real or imagined abandonment, as the potential behavioral outcomes of these dispositions. In other words, whereas in the traditional approach such symptoms constitute the PD itself, in the new model they could be seen as potential outcomes of the underlying PD. The Alternative Model of Personality Disorders (AMPD) in Section III of the DSM-5 frames the core of PDs as a range of impairments in self- and interpersonal functioning, that may underlie typical symptoms or disabilities. Taking this perspective, impaired personality functioning may be considered as a mediator between childhood maltreatment and potential symptomatic sequelae. This matches a developmental perspective assuming that personality functioning refers to the development of certain abilities, like the ability to self-reflect, to regulate emotions, to attune to the mind of others, to experience safety within intimate relationships and to design a sense of uniqueness and self-direction [14]. PDs then reflect the impaired development of these abilities, serving as a risk disposition for developing mental health problems. In fact, individuals with impaired personality functioning have found to be at increased risk for depression and anxiety disorders [15–17]. In addition, a study among 228 psychiatric outpatients and incarcerated addicts showed that impaired personality functioning was significantly associated with lower healthy functioning, fulfillment, and well-being in adulthood [18]. Personality functioning could, thus indeed, be conceptualized as one of the many aspect of resilience mediating the pathogenic impact of childhood maltreatment.

Previous work has, actually, shown that several facets of personality functioning may serve as a mediator for the long-term consequences of childhood maltreatment. For instance, the pathogenic effects of childhood maltreatment have been demonstrated to be mediated by low self-esteem [19], negative self-associations [20], self-compassion and shame [21], emotion dysregulation [22–24], mentalizing incapacity [25], attachment [26], self-blame, and interpersonal difficulties [27]. In a study among 235 pregnant women and 66 expecting fathers, Berthelot et al. [28] found that the association between childhood maltreatment and psychological symptoms during pregnancy was partially mediated by the level of reflective functioning. Moreover, the capacity to self-reflect also predicted parents’ feelings of competence related to parenthood and their psychological investment in the unborn child. The authors conclude that reflective functioning may, therefore, serve as an important aspect of resilience mitigating the aversive impact of parental trauma. Similarly, London et al. [29] demonstrated the mediating role of attachment insecurity in the association between exposure to violence and experiencing symptoms of Post-Traumatic Stress Disorder (PTSD) in adolescents. Huang et al. [30] studied the mediating role of mentalizing and attachment in a sample of 184 PD patients and 111 community controls. They found that lower mentalizing ability and attachment insecurity mediated the link between childhood maltreatment and PTSD symptoms. While this is just a brief snapshot of relevant findings, they all seem to converge in that certain processes of emotion regulation, self-direction, social cognition, threat recognition, and interpersonal support may mediate the pathogenic impact of childhood maltreatment and explain the development of transdiagnostic psychopathological expressions [31]. The previously mentioned AMPD may provide a conceptual framework for the abilities that may be relevant to understand the mediating role of general personality functioning, with the Level of Personality Functioning Scale (LPFS) providing a generalized dimension of severity that encompasses aspects like self-functioning (i.e., self-reflection, emotion regulation, and self-direction) and interpersonal functioning (i.e., social cognition, empathy, and interpersonal security).

To the best of our knowledge, only three studies investigated the mediating effect of personality functioning as such in the association between childhood maltreatment and psychopathology so far. While the study of Dagnino et al. [16] found significant mediating effects of personality functioning between physical and sexual abuse and depressive symptomatology, the study of Freier et al. [32], revealed that up to two-thirds of the associations between different types of childhood maltreatment and symptoms of depression and anxiety were mediated by impaired personality functioning. The study of Krakau et al. [33], moreover, revealed that identity perception and self-reflective capacities had the strongest mediating impact between overall childhood maltreatment and mental distress. Thus, there is, indeed, some evidence that the association between childhood maltreatment and psychopathology may be mediated by personality functioning and that self-functioning may have the strongest mediating effect. However, current findings either result from community-based or clinical settings and are either based on childhood maltreatment screening instruments or are limited to general personality functioning. In addition, current findings are entirely based on self-reported personality functioning, according to the operationalized psychodynamic diagnosis structure questionnaire (i.e., OPD Structure Questionnaire [OPD-SQ]; [34, 35]). The sole use of self-report to assess personality impairments has, however, been questioned [36]. A study in a high-risk sample with detailed measures of childhood maltreatment and self-reported mental health problems, investigating different domains of impaired personality functioning with a clinical interview according to the AMPD, could, thus, extend current evidence.

The aim of the present study was to examine impaired personality functioning as a potential mediator between different types of childhood maltreatment and self-reported mental health problems in young adults with a history of residential child welfare and/or juvenile justice placements. In addition, this study sought to identify the domains of impaired personality functioning that have the strongest mediating effects between different types of childhood maltreatment and mental health problems. Based on the aforementioned findings, we postulated that young adults with a history of residential child welfare and juvenile justice placements may show higher levels of impaired personality functioning when facing childhood maltreatment, resulting in a greater severity of self-reported mental health problems. Specifically, we hypothesized: 1) that different types of childhood maltreatment (i.e., emotional neglect, physical neglect, emotional abuse, physical abuse, and sexual abuse) would be positively associated with mental health problems, 2) that these effects would be partially mediated by impaired personality functioning and 3) that abilities related to self-functioning would mediate these effects more strongly than abilities related to interpersonal functioning.

## Methods

### Study design

Data was obtained from the longitudinal Swiss study “Youth Welfare Trajectories: Learning from Experiences” (German: Jugendhilfeverläufe: Aus Erfahrung lernen [JAEL] [37]), a 10-year follow-up study of the “Swiss Study for Clarification and Goal-Attainment in Child Welfare and Juvenile Justice Institutions” (German: Modellversuch Abklärung und Zielerreichung in stationären Massnahmen [MAZ.] [38]). The baseline study was conducted between 2007 and 2011 with the primary aims of (1) describing the mental health of children and adolescents in residential care and (2) investigating the outcomes of residential youth care over an approximately 1-year period. Child welfare and juvenile justice institutions accredited by the Swiss Federal Ministry of Justice were invited to participate, of which 64 institutions agreed to take part. Juveniles who had been living for at least 1 month in one of these 64 included institutions and had sufficient language skills in German, French, or Italian as well as sufficient intelligence scores (IQ > 70) were eligible for participation. Overall, 592 children and adolescents aged 6–26 years (mean age = 16.3 years) participated at baseline. After a follow-up period of approximately 10 years, participants were reassessed between 2018 and 2020 with the aim of investigating their psychosocial development and their transition out of care. Of the 511 participants who initially agreed to be contacted for a possible follow-up at baseline, 231 (45.2%) participated in the follow-up study. Despite considerable efforts, 8 (1.6%) participants could not be found, 121 (23.7%) did not respond to our contact request, 99 (19.4%) refused to participate, 44 (8.6%) agreed to participate, but eventually did not fill out the informed consent form or any questionnaire, and 8 (1.6%) were deceased. A study flow-chart is provided in Additional file 1: Figure S1. An analysis of the sample attrition showed no significant differences in sociodemographic features at baseline (i.e., age, gender, number of former placements, average duration in residential care) between the participants who took part in the follow-up and those who did not. The follow-up assessment consisted primarily of a set of online questionnaires that participants could complete from home. Participants were then invited to a face-to-face meeting, where they were reassessed using semi-structured clinical interviews and semi-structured qualitative in-depth interviews regarding mental health, psychosocial problems, and offending behavior. Assessment was conducted by trained psychologists, doctoral students, and research assistants. The study procedure was approved by the Ethics Committee Northwestern and Central Switzerland (EKNZ, Ref.: 2017-00718).

### Participants

As the primary aim of this study was to investigate the mediating role of personality functioning between childhood maltreatment and mental health problems, only participants with complete data from the Childhood Trauma Questionnaire—Short Form (CTQ-SF; [39]), the Semi-structured Interview for Personality Functioning DSM-5 (STiP-5.1; [40]), as well as the self-report questionnaires of the Achenbach System of Empirically Based Assessment (ASEBA; [41]) were included in the analyses. The final sample included 173 participants (32.76% female) with a mean age of 26.61 (*SD* = 3.27; range 18–38 years) (Table 1). Participants that were excluded from the current analyses were slightly younger than included participants (mean age = 24.81; *SD* = 3.79; *t*(79) = 2.12; *p* = 0.037). No statistically significant differences were found in gender (χ^2^(1) = 0.000; *p* = 1.000), number of placements (*t*(98) = 0.90; *p* = 0.367), average duration in residential care (*t*(49) = 0.85; *p* = 0.401), personality functioning (*t*(5) =—0.55; *p* = 0.606), and mental health problems (*t*(48) =—1.17; *p* = 0.247).

**Table 1:** Sample characteristics (N = 173)

	*M* (*SD*)
Age (years)	26.60 (3.28)
Number of placements in residential care	3.70 (3.26)
Average duration in residential care (years)	6.99 (5.34)
	*n* (%)
Gender (female)	57 (32.95)
Childhood maltreatment	132 (76.30)
One type of childhood maltreatment	30 (17.34)
Two types of childhood maltreatment	45 (26.01)
> Three types of childhood maltreatment	57 (32.95)
Emotional abuse	44 (25.43)
Physical abuse	58 (33.53)
Sexual abuse	35 (20.23)
Emotional neglect	105 (60.69)
Physical neglect	87 (50.29)
Current mental-health problems	
Overall mental health problems	53 (30.6)
Internalizing problems	51 (29.5)
Externalizing problems	42 (24.3)
Current mental-health treatment	41 (23.7)

### Measurements

#### Sociodemographic characteristics

Sociodemographic information—age, gender, number of placements, average duration in residential care (i.e., total time spent in residential care and/or juvenile justice institutions) and current mental health treatment—was assessed using a computer-based questionnaire.

#### Childhood maltreatment

Childhood maltreatment was measured retrospectively at follow-up, with the Childhood Trauma Questionnaire—Short Form (CTQ-SF; [39]). The CTQ-SF is a self-report questionnaire, consisting of 25 retrospective items assessing childhood maltreatment histories, each scored on a 5-point Likert scale (i.e., “never true” to “very often true”). Three additional minimalization/denial items are used to identify individuals who may be underreporting traumatic events. The CTQ-SF includes five subscales: emotional abuse, physical abuse, sexual abuse, physical neglect, and emotional neglect. The individual items are summed to give subscale scores from 5 to 25, as well as a weighted total score, which is calculated based on the score of each subscale adjusted for the number of items included in that subscale. The CTQ-SF was found to show high reliability and validity, with intraclass correlation coefficients ranging from r = 0.76–0.86 [39].

#### Personality functioning

Personality functioning was assessed with the Semi-structured Interview for Personality Functioning DSM-5 (STiP-5.1; [40]). The STiP-5.1 is a clinician-rated interview, assessing the overall level of personality functioning according to the Alternative Model of Personality Disorders (AMPD), introduced in Section III of the DSM-5. The interview consists of 28 open questions and several optional clarifying questions, divided into two main domains of personality functioning: self-functioning and interpersonal functioning. Self-functioning, on the one hand, refers to a range of adaptive abilities related to the following two subdomains: identity (i.e., experience of oneself as unique, the stability of self-esteem, and the capacity for emotion regulation) and self-direction (i.e., the pursuit of meaningful goals, the utilization of prosocial internal standards of behavior, and the ability to productively self-reflect). Interpersonal functioning on the other hand, refers to abilities of the two subdomains: empathy (i.e., ability to understand others’ experiences and motivations, to tolerate differing perspectives, and to understand the impact of one's behavior on others) and intimacy (i.e., the ability to establish durable and meaningful relationships, to experience and tolerate closeness, and mutual regard). Each subdomain relates to three abilities derived from the LPFS, resulting in a total of 12 facets, rated each on a 5-point scale: Level 0 (little or no impairment), Level 1 (some impairment), Level 2 (moderate impairment), Level 3 (severe impairment), and Level 4 (extreme impairment). The final STiP-5.1 score can either consist of a total score related to the global level of personality functioning or four domain scores related to the four subdomains (i.e., identity, self-direction, empathy, intimacy). For the present analyses, we combined the 12 facets scores to obtain an overall dimensional score with the widest possible range of scores. The STiP-5.1 presents high internal consistency, with a Cronbach's α of 0.97 for the total scale. The interrater reliability is shown to be good, with ICCs ranging from 0.81 to 0.92 in an overall sample and from 0.58 to 0.80 in a clinical sample [40].

#### Mental health problems

Mental health problems were assessed using the self-report questionnaires of the Achenbach System of Empirically Based Assessment scales (ASEBA; Youth Self-Report [YSR; [42]]; Young Adult Self-Report [YASR; [43]]; Adult Self-Report [ASR; [44]]. The YSR (i.e., 118 items), YASR (i.e., 124 items) and ASR (i.e., 120 items) are designed to assess emotional and behavioral problems in adolescents (11–18 years), young adults (i.e., 18–30 years) and adults (i.e., 18–59 years). Each item is rated on a three-point Likert scale (0 = not true, 1 = sometimes true, 2 = very true). Summing the scores of the eight subscales results in a total score, as well as two superordinate scores for internalizing and externalizing symptoms. In the current study, raw scores were transformed into t-scores, with a t-score  ≥ 60 considered to be clinically relevant.

### Statistical analysis

First, descriptive statistical analyses were calculated for sociodemographic variables, childhood maltreatment, personality functioning and mental health problems. Second, Pearson’s correlation coefficients were calculated to investigate the associations between childhood maltreatment and mental health problems (i.e., hypothesis 1). Third, mediation analyses were conducted, using structural equation modeling adjusted for age and gender, in order to explore the mediating role of impaired personality functioning between childhood maltreatment and mental health problems (i.e., hypotheses 2 and 3). Mediation analyses seek to determine the extent to which the effect of an exposure (i.e., childhood maltreatment) on an outcome variable (i.e., mental health problems) is mediated by an intermediate variable (i.e., personality functioning). The mediation effect is referred to as the indirect effect, while the portion of the exposure that does not go through the mediating variable is referred to as the direct effect. Summing up the direct and indirect effect results in the total effect of an exposure (i.e., childhood maltreatment) on the outcome (i.e., mental health problems). We, first, calculated the indirect effect of impaired personality functioning (i.e., STiP-5.1 total score) between different types of childhood maltreatment (i.e., CTQ-SF total score, emotional abuse, physical abuse, sexual abuse, physical neglect, and emotional neglect) and mental health problems to test hypothesis 2 (i.e., impaired personality functioning significantly mediates the association between different types of childhood maltreatment and mental health problems). We, then dropped the STiP-5.1 total score as potential mediator and simultaneously incorporated the STiP-5.1 domains self-functioning and interpersonal functioning to test hypothesis 3 (i.e., self-functioning mediates the effect of childhood maltreatment on mental health problems more strongly than interpersonal functioning). The proportion of the mediating effect indicates the proportion of the total effect that occurs through the mediating effect (i.e., indirect effect). Based on recommendations by Hayes [45], a bootstrapping sampling procedure with 5′000 bootstrapped samples was applied in the structural equation models. Bootstrapping is a nonparametric approach that accounts for non-normal distribution and provides nonbiased confidence intervals [46] that allow more accurate inferences when the sample size is small. According to Preacher et al. [47] mediation emerges, when the mediating effect is found to be significant and if zero is not included in the 95% confidence interval. All effects were adjusted for age and gender. Multicollinearity of independent variables was not considered to be an issue (see Additional file 1: Table S1). All statistical analyses were conducted using RStudio (Version 1.4.1106; [48]). Statistical significance was set to *p* < 0.05. Complete case analyses were performed.

## Results

### Descriptive characteristics

Findings on the descriptive analyses are presented in Table 1. Participants spent an average of 6.99 years (*SD* = 5.34) in the child welfare and/or juvenile justice system, with a mean number of 3.70 (*SD* = 3.26) placements. Overall, 76.3% (N = 132) participants indicated at least one type of childhood maltreatment, with 32.95% (N = 57) reporting even three or more. Emotional neglect was most commonly reported (60.69%), followed by physical neglect (50.29%) and physical abuse (33.53%). A total of 30.6% (N = 53) participants self-reported mental health problems, with internalizing problems (29.5%) being slightly more often reported than externalizing problems (24.3%). 23.7% (N = 41) participants reported current mental health treatment.

### Personality functioning

Findings regarding the level of personality functioning are presented in Table 2. Overall, 33.52% (N = 58) participants showed significant impairments in personality functioning, with 20.93% (N = 36) exhibiting moderate, 11.05% (N = 19) severe and 1.74% (N = 3) extreme impairments. A total of 27.74% (N = 48) participants exhibited impairments in Self-functioning. Of these, 26.01% (N = 45) showed impairments in Identity and 23.12% (N = 40) showed impairments in Self-direction. A total of 25.43% (N = 44) participants exhibited impairments in Interpersonal functioning, with 21.96% (N = 38) showing impairments in Empathy and 18.50% (N = 32) showing impairments in Intimacy.

**Table 2:** Personality Functioning (N = 173)

	No to low impairment	Moderate impairment	Severe impairment	Extreme impairment
	n (%)	n (%)	n (%)	n (%)
Overall personality functioning	115 (66.47)	36 (20.93)	19 (11.05)	3 (1.74)
Self-functioning	125 (72.25)	27 (15.70)	19 (11.05)	2 (1.16)
Identity	128 (73.99)	25 (14.37)	19 (11.05)	1 (0.57)
Self-direction	133 (76.88)	20 (11.49)	15 (8.62)	5 (2.87)
Interpersonal functioning	129 (74.57)	29 (16.76)	13 (7.51)	2 (1.16)
Empathy	135 (78.03)	27 (15.52)	11 (6.32)	0 (0.00)
Intimacy	141 (81.50)	19 (10.98)	9 (5.20)	4 (2.31)

### Associations between childhood maltreatment, personality functioning and mental health problems

Findings regarding the associations between childhood maltreatment, personality functioning, and mental health problems are presented in Table 3. The strongest association was found between personality functioning and mental health problems (r = 0.36, *p* < 0.001), indicating that the greater the impairments in personality functioning, the greater the mental health problems. All types of childhood maltreatment were positively associated with mental health problems, ranging from r = 0.16 (i.e., sexual abuse) to r = 0.34 (i.e., emotional abuse), suggesting that more severe childhood maltreatment led to more severe mental health problems. Finally, overall childhood maltreatment (r = 0.23, *p* = 0.002), emotional neglect (r = 0.17, *p* = 0.027), emotional abuse (r = 0.19, *p* = 0.013), and sexual abuse (r = 0.18, *p* = 0.019) were positively associated with personality functioning, indicating that more severe forms of these types of childhood maltreatment significantly led to more severe impairments in personality functioning.

**Table 3:** Associations between childhood maltreatment, personality functioning and mental health problems (N = 173)

	Personality functioning	Mental health problems
Overall childhood maltreatment	0.23^**^	0.34^***^
Emotional neglect	0.17^*^	0.28^***^
Physical neglect	0.12	0.18^*^
Emotional abuse	0.19^*^	0.34^***^
Physical abuse	0.13	0.19^*^
Sexual abuse	0.18^*^	0.16^*^
Personality functioning	–	0.36^***^

Note. Pearson’s r are reported. ^*^p < 0.05; ^**^p < 0.01; ^**^*p < 0.001

### Mediation analyses

**Fig. 1:**
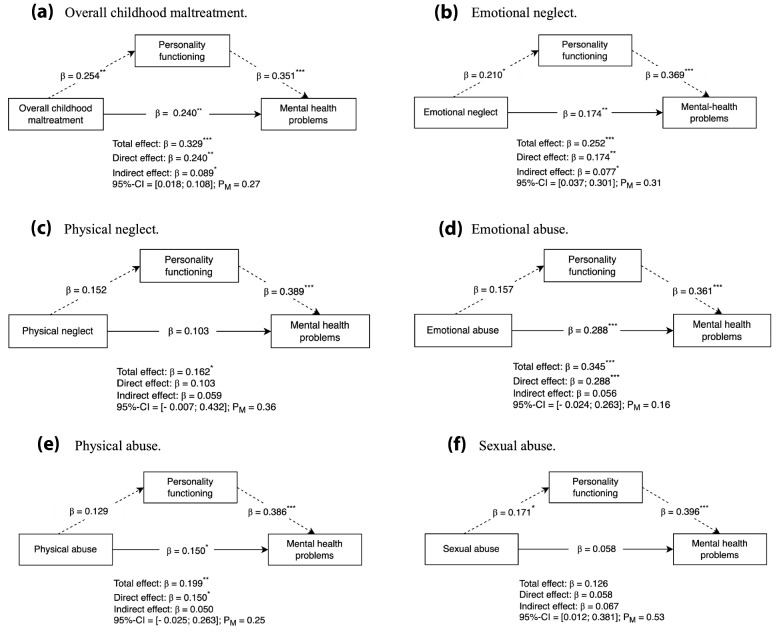
Mediating model of personality functioning in the association between childhood maltreatment and adult mental health problems. **a.** Overall childhood maltreatment. **b.** Emotional neglect. **c.** Physical neglect. **d.** Emotional abuse. **e.** Physical abuse. **f.** Sexual abuse. Standardized β-coefficients, adjusted for age and gender are reported. For convenience, indirect effects are denoted by dotted lines. 95%-CI 95%-Confidence interval; P_M_ = Proportion of the mediating effect as proportion of the total effect. ^*^p < 0.05; ^**^p < 0.01; ^***^p < 0.001

Findings regarding the mediating effect of impaired personality functioning between different types of childhood maltreatment and mental health problems are presented in Fig. 1. First, overall childhood maltreatment, emotional neglect and sexual abuse significantly predicted impaired personality functioning (*β* = 0.254, *p* = 0.004; β = 0.210, *p* = 0.010; and *β* = 0.171, *p* = 0.043 respectively). Second, personality functioning significantly predicted mental health problems for all types of childhood maltreatment. Third, significant total effects were found for all types of childhood maltreatment, except for sexual abuse. Fourth, personality functioning revealed significant indirect effects (i.e., mediating effects) for overall childhood maltreatment (*β* = 0.089; *p* = 0.008) and emotional neglect (*β* = 0.077; *p* = 0.016). The proportion of indirect effects of the total effect was 27% for overall childhood maltreatment and 31% for emotional neglect. This indicates that about one-third of the association between overall childhood maltreatment and mental health problems, as well as emotional neglect and mental health problems was mediated through impaired personality functioning. Fifth, significant direct effects remained for overall childhood maltreatment (*β* = 0.240; *p* = 0.001), emotional neglect (*β* = 0.174; *p* = 0.009), emotional abuse (*β* = 0.288; *p* < 0.001) and physical abuse (*β* = 0.150; *p* = 0.018).

**Fig. 2:**
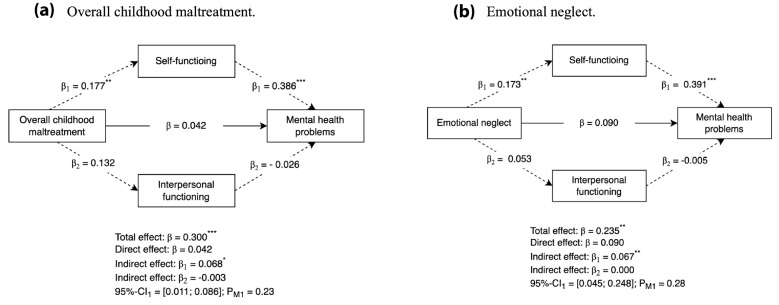
Mediating model of self-functioning and interpersonal functioning in the association between childhood maltreatment and adult mental health problems. **a.** Overall childhood maltreatment. **b.** Emotional neglect. Standardized β-coefficients, adjusted for age and gender are reported. For convenience, indirect effects are denoted by dotted lines. 95%-CI 95%-Confidence interval; P_M_ = Proportion of the mediating effect as proportion of the total effect. ^*^p < .05; ^**^p < .01; ^***^p < .001

As impaired personality functioning was not found to significantly mediate the association between emotional abuse, physical abuse and neglect, and sexual abuse and mental health problems, hypothesis 3 was only tested for overall childhood maltreatment and emotional neglect. The findings are presented in Fig. 2. First, overall childhood maltreatment as well as emotional neglect significantly predicted self-functioning (*β*_*1*_ = 0.177, *p* = 0.007; and *β*_*1*_ = 0.173, *p* = 0.003 respectively) but not interpersonal functioning. Second, only self-functioning significantly predicted mental health problems for both types of childhood maltreatment (i.e., *p* < 0.001). Third, significant total effects were found for both types of childhood maltreatment (i.e., *p* < 0.001). Fourth, only self-functioning revealed a significant indirect effect (i.e., mediating effect) for overall childhood maltreatment (*β* = 0.068; *p* = 0.015) and emotional neglect (*β* = 0.067; *p* = 0.008). The proportion of the indirect effect of the total effect was 23% for overall childhood maltreatment and 28% for emotional neglect. This indicates that almost one-quarter of the association between overall childhood maltreatment and mental health problems, and almost one-third of the association between emotional neglect and mental health problems was mediated through self-functioning. Fifth, no significant direct effects remained when including both, self-functioning, and interpersonal functioning as mediators in the association between overall childhood maltreatment, emotional neglect, and mental health problems.

## Discussion

The aim of the current study was to examine impaired personality functioning as a potential mediator between different types of childhood maltreatment and mental health problems in young adults with a history of residential child welfare and/or juvenile justice placements. In addition, this study sought to identify domains of impaired personality functioning that have the strongest mediating effect between different types of childhood maltreatment and mental health problems. The current results revealed at least three major findings to be discussed below.

First, as expected, a significant positive association was found between different types of childhood maltreatment and mental health problems, indicating childhood maltreatment to increase the risk for higher levels of overall psychopathology. This is in line with our first hypothesis, suggesting that more severe childhood maltreatment leads to substantially higher levels of internalizing and externalizing symptoms [16, 32, 33]. Notably, emotional abuse and neglect showed the largest associations with mental health problems. Although there is considerable support for the other types of childhood maltreatment [49, 50], our findings add to the growing literature highlighting the strong pathogenic effects of emotional abuse and neglect [51–54]. Thus, not only the more obvious types of childhood maltreatment (i.e., physical abuse and neglect, and sexual abuse) tend to have a significant impact on mental health, rather, the more subtle and often hidden forms of childhood maltreatment, such as emotional abuse and neglect, might lead to even higher levels of mental health problems [55].

Second, and partially in line with hypothesis 2, our findings revealed impaired personality functioning to be a significant mediator between overall childhood maltreatment and mental health problems. This is consistent with previous findings [16, 32, 33], suggesting a continuous process, in which childhood maltreatment deteriorates personality functioning, which, in turn, leads to higher levels of mental health problems. When considering different types of childhood maltreatment, impaired personality functioning, however, was found to be a significant mediator only for emotional neglect. This finding is inconsistent with hypothesis 2, and somewhat surprising, given that the study from Freier et al. [32] found a significant mediating effect for all types of childhood maltreatment. The findings from Freier et al. [32], however, resulted from a large community-based sample and prevalence rates of childhood maltreatment were substantially lower compared to our sample. In addition, participants in our sample, were exposed to a range of other significant risk-factors—such as unfavorable parenting practices, low socioeconomic status, parental mental disorders, early mental health problems, self-harming behavior, psychopathic traits, and youth delinquency—all of which may have shaped personality functioning, besides traumatic experiences. Moreover, emotional neglect predicted impaired personality functioning more strongly than other types of childhood maltreatment in our sample. This is in line with findings from a clinical sample from Gander et al. [56], who found that emotional abuse and neglect were twice as strongly related to impaired personality functioning than physical abuse, physical neglect, and sexual abuse. Our findings, thus, support the growing evidence that emotional neglect may be more relevant in the context of personality functioning than physical neglect, physical abuse, and sexual abuse [57]. Nevertheless, the total mediating effect of impaired personality functioning only accounted for about 30% of the total effect between overall childhood maltreatment, emotional neglect, and mental health problems, meaning that 70% still proceeded through the direct effect from childhood maltreatment to mental health problems. Yet it may be that an additional proportion was referred by other potential mediators, such as the parent–child relationship [58], physical exercise [59], maladaptive coping strategies [60], brain alterations [61], and verbal abilities [62], which all have been found to significantly mediated the association between childhood maltreatment and mental health problems. This highlights the crucial need to conduct further studies with concurrently different mediators.

Third, and partially consistent with hypothesis 3, our findings revealed a significant mediating effect of impaired self-functioning when both self-functioning and interpersonal functioning were included as potential mediators in the relationship between overall childhood maltreatment and emotional neglect. This is in line with the findings from Krakau et al. [33], indicating a pronounced impact of identity perception and self-direction in mediating between childhood maltreatment and mental health problems. This supports, in addition, previous findings that found negative self-associations [20, 59], impaired self-compassion and shame [21], negative self-efficacy [63] and impaired reflective functioning [64] to be significant mediators between childhood maltreatment and mental health problems. Indeed, childhood maltreatment, particularly emotional neglect [65], has been repeatedly shown to profoundly affect self-identity across the lifespan [66]. Unlike emotional abuse, which involves the presence of unexpected negative inputs, emotional neglect, involves an absence of expected positive inputs [67–69], or simply the absence of any input. This lack of responsiveness to a child’s needs may compromise their ability to identify and value their own feelings and needs, which in turn, may lead to a lack of clarity about their own identity and self-direction [65]. This might explain, at least in part, why only impaired self-functioning significantly mediated the association between emotional neglect and mental health problems, when both self-functioning and interpersonal functioning were included as potential mediators. Yet again, the mediating effect of self-functioning only accounted for about 25% of the total effect between overall childhood maltreatment, emotional neglect, and mental health problems, meaning that 75% still proceeded through the direct effect from childhood maltreatment to mental health problems as well as potentially other mediators.

### Strengths

The present study contributes to current research on the association between childhood maltreatment and mental health problems by explicitly presenting findings from a high-risk sample. Only a few studies have investigated the mediating role of impaired personality functioning as a potential mediator between childhood maltreatment and mental health problems, and to the best of our knowledge, none have yet investigated this effect in a high-risk sample. Yet, children and adolescents placed in the residential child welfare and/or juvenile justice system have a particularly high risk of developing impaired personality functioning as well as mental health problems due to a cumulation of risk factors (i.e., childhood maltreatment, unfavorable parenting practices, low socioeconomic status, childhood psychopathology, self-harming behavior, and youth delinquency), which is why such samples provide particularly valuable insights into the association between childhood maltreatment and mental health problems. The inclusion of different types of childhood maltreatment further allowed to examine which types of childhood maltreatment are mostly mediated by impaired personality functioning. Finally, by simultaneously including self-functioning and interpersonal functioning as potential mediators, we were able to differentiate the mediating role of two distinct domains of personality functioning.

### Limitations

Nonetheless, current findings must be interpreted under the consideration of some limitations. First, the use of mediation analysis on cross-sectional data has widely been questioned as cross-sectional estimates can either seriously under- or overestimate indirect effects [70]. The present mediation analyses were, however, conducted according to Hayes et al. [45] as an attempt to test a specific model. Therefore, findings must be interpreted with caution and further investigations, using longitudinal studies, are highly needed. Second, findings on childhood maltreatment relied entirely on retrospective self-reports, which might result in recall bias [71]. In addition, retrospective reports could be affected by personality functioning and/or actual functioning. However, the CTQ has found to be valid [72], and no significant difference between prospective and retrospective self-reports of childhood maltreatment have been found in a comparative study [73]. Third, mental health problems were assessed using self-report questionnaires, making responses susceptible to various forms of biases, such as social desirability and limited self-awareness [74]. Fourth, the current study did not consider possible moderators of childhood maltreatment, such as age at the time of maltreatment, frequency, and duration of maltreatment as well as the perpetrator relationship, all of which have been found to considerably affect the risk for psychopathology. As such, exposure to abuse at an earlier age is more likely to result in higher levels of psychopathology, earlier onset, higher number of comorbidities and poorer treatment outcomes [75]. Including such moderators could, thus, provide valuable insight into the relationship between childhood maltreatment and mental health problems. Finally, as maltreatment often extends throughout childhood and adolescence, a developmental cascade model and potential sensitive periods for influences of maltreatment and personality functioning should be explored within future studies.

### Implications

For clinical practice, the current findings indicate that children and adolescents involved in the child welfare and/or juvenile justice system, should be systematically assessed for childhood maltreatment, personality functioning and mental health problems, as prevalence rates are distressingly high. In addition, the findings emphasize the need to sensitize standard mental health treatments to childhood maltreatment and impaired personality functioning. In terms of trauma-informed practices, mental health services should provide a broad-based understanding for childhood maltreatment and the pathways in which childhood maltreatment may affect the development of mental abilities like emotion regulation, self-reflection, and social cognition and, therefore, may lead to maladaptive coping strategies and problematic behavior. In addition, mental health services should provide safe, trusting, and continuous nurturing relationships, in order to promote resilience in maltreated children and adolescents [76, 77]. Moreover, mental health services should assist vulnerable children and adolescents in developing these mental abilities. While this goes beyond providing adequate care, it requires a sensitive relationship which is attuned to the personal needs and emotions of these children. In terms of personality functioning, emerging evidence suggests the use of severity- and trait-informed treatment methods. In addition, the different facets described by the AMPD or ICD-11, may help clinicians to identify individual problems across domains (e.g., identity, self-reflection, emotion regulation, and interpersonal security), resulting in more tailor-made treatments [78]. Combined with trauma-informed practices, such interventions could help maltreated children and adolescents to develop more adaptive self-concepts, self-direction, and emotion regulation capacities, which, in turn, could potentially mitigate psychopathological outcomes. As childhood maltreatment is, however, neither a necessary nor a sufficient condition for developing mental health problems and, likewise, does not necessarily compromise personality functioning, future research should focus on resilience to promote healthy development in maltreated children and adolescents. In addition, it is important to bear in mind that personality functioning only accounts for a small part of the pathogenic impact of maltreatment, thus, further investigations are highly needed to focus on other potential mediators, as for instance, parent–child relationship, physical exercise, and brain alterations, which all have been found to significantly mediated the association between childhood maltreatment and mental health problems [58, 59, 61]. Furthermore, age, gender, socioeconomic status, and current mental health treatment were identified as important factors affecting the results of this study. Future research should, therefore, investigate the impact of these factors on personality functioning to further explore how each factor affects the long-term consequences of childhood maltreatment. Finally, childhood neglect has been the most overlooked and least researched form of childhood maltreatment [79, 80], which may be referred to as the “neglect of neglect” [81–83]. This lack of research is partly due to insufficient measurement instruments to assess childhood neglect. Thus, future research should investigate neglect on its own right and develop appropriate measurements.

## Conclusion

The present findings add to the current understanding of impaired personality functioning, in particular impaired self-functioning, as an important mediator of the association between overall childhood maltreatment, emotional neglect and mental health problems. The findings, thus, indicate that emotional neglect may be particularly important in the context of childhood maltreatment, personality functioning, and mental health problems and, therefore, should not be overlooked next to the more “obvious” forms of childhood maltreatment. Future research should address the sequalae of childhood maltreatment in high-risk samples, particular prone to the adverse consequence of childhood maltreatment, including concurrently different mediators, in order to unpack the complex association between childhood maltreatment and mental health problems. Combining interventions designed for personality functioning with trauma-informed practices in standard mental health services might foster resilience and counteract the psychopathological outcomes of maltreated children and adolescents.

## Supplementary Information

**Additional file 1: Figure S1.** Flow-chart of the study sample. **Table S1**. Multicollinearity of independent variables (Pearson’s r).

## Data Availability

The raw data supporting the conclusions of this article can be requested from the first author.
