# Supplementary material for: Personality functioning and the pathogenic effect of childhood maltreatment in a high-risk sample
Source: Child Adolesc Psychiatry Ment Health. 2022 Nov 30;16:95. doi: 10.1186/s13034-022-00527-1 (PMC9710065; doi:10.1186/s13034-022-00527-1)
Supplement: Supplementary file 1 — Additional file 1: Figure S1. Flow-chart of the study sample. Table S1. Multicollinearity of independent variables (Pearson’s r). [file 13034_2022_527_MOESM1_ESM.docx]

Supplementary Material

Baseline sample
(*N* = 592)

**Baseline (t0)**

Reason for drop-out (*N* = 81):

- Did not provide informed consent for a possible follow-up (*N* = 81)

Eligible for follow-up
(*N* = 511)

Reason for drop-out (*N* = 280):

- Could not be located (*N* = 8)
- Could not be reached (*N* = 121)
- Refused to participate (*N* = 99)
- Did not provide informed consent (N = 44)
- Were deceased (*N* = 8)

Follow-up sample
(*N* = 231)

**Follow-Up (t2)**

Reason for drop-out (*N* = 58):

- Only online assessment available (*N* = 51)
- Missing data on different variables (*N* = 7)

Sample used for analyses

(*N* = 173)

**Figure S1.** Flow-chart of the study sample.

**Table S1**. Multicollinearity of independent variables (Pearson’s r).

|  | 1. | 2. | 3. | 4. | 5. | 6. | 7. | 8. | 9. |
| --- | --- | --- | --- | --- | --- | --- | --- | --- | --- |
| 1. CTQ-SF total score |  |  |  |  |  |  |  |  |  |
| 1. Emotional neglect | 0.74^***^ |  |  |  |  |  |  |  |  |
| 1. Physical neglect | 0.67^***^ | 0.61^***^ |  |  |  |  |  |  |  |
| 1. Emotional abuse | 0.86^***^ | 0.55^***^ | 0.49^***^ |  |  |  |  |  |  |
| 1. Physical abuse | 0.71^***^ | 0.28^**^ | 0.29^***^ | 0.58^***^ |  |  |  |  |  |
| 1. Sexual abuse | 0.41^***^ | 0.04 | - 0.04 | 0.25^*^ | 0.19 |  |  |  |  |
| 1. STiP-5.1 facet sum score | 0.20 | 0.19 | 0.16 | 0.11 | 0.11 | 0.11 |  |  |  |
| 1. Self-functioning | 0.20 | 0.20 | 0.15 | 0.14 | 0.11 | 0.09 | 0.84^***^ |  |  |
| 1. Interpersonal functioning | 0.20 | 0.13 | 0.08 | 0.15 | 0.10 | 0.23 | 0.76^***^ | 0.61^***^ |  |
| 1. ASEBA total score | 0.34^***^ | 0.28^**^ | 0.18 | 0.34^***^ | 0.19 | 0.16 | 0.38^***^ | 0.42^***^ | 0.28^**^ |

Note. CTQ -SF= Childhood Trauma Questionnaire – Short Form; STiP-5.1 = Semi-structured Interview for Personality Functioning DSM-5.
